# A Hybrid Approach for Predicting PM_2.5_ Exposure: van Donkelaar et al. Respond

**DOI:** 10.1289/ehp.1002706R

**Published:** 2010-10

**Authors:** Aaron van Donkelaar, Randall Martin, Caroline Verduzco, Mike Brauer, Ralph Kahn, Robert Levy, Paul Villeneuve

**Affiliations:** Department of Physics and Atmospheric Science, Dalhousie University, Halifax, Nova Scotia, Canada, E-mail: Aaron.van.Donkelaar@dal.ca; School of Environmental Health, University of British Columbia, Vancouver, British Columbia, Canada; NASA Goddard Space Flight Center, Greenbelt, Maryland; Population Studies Division, Health Canada, Ottawa, Ontario, Canada

We thank Kumar for his comments on our article (van Donkelaar et al. 2010). We agree that integration of satellite-based aerosol optical depth (AOD) with a chemical transport model (CTM) is valuable to develop estimates of air quality. We also agree that despite the major recent advancements in remote sensing and CTMs, further development of these methods would continue to improve the estimates of fine particulate matter [< 2.5 μm in aerodynamic diameter (PM_2.5_)]. We are grateful for the opportunity to expand on those issues here.

As pointed out by Kumar, the relationship between ground-level PM_2.5_ and AOD is complex, with dependence on the scattering properties of the local aerosol (a function of aerosol type and atmospheric conditions) and their vertical distribution (a function of boundary layer height, transport, production, and loss). These factors include effects of atmospheric pressure and surface concentration. The method we used in our study (van Donkelaar et al. 2010) was designed specifically to account for all of these factors (not only relative humidity, as implied by Kumar). η is defined as the ratio of surface PM_2.5_ to total column AOD, where the definition of PM_2.5_ is at either 35% or 50% relative humidity, in accordance with regional ground measurement standards, and total column AOD includes the effects of local relative humidity on aerosol extinction.

We agree that higher resolution calculations of η would continue to improve the PM_2.5_ estimates and are actively developing this capability. However, it is worth clarifying that the long (~ 1 week) aerosol lifetime does not detract from, but rather it contributes to the accuracy of a simulation at 2° × 2.5°. Short-lived species (< 1 day) have more subgrid spatial variation due to the effects of more rapid atmospheric losses. The smoothing associated with longer-lived aerosols enables a global model to sufficiently capture major processes affecting η.

A number of promising developments are also occurring in satellite remote sensing. The Deep Blue algorithm (Hsu et al. 2006) noted by Kumar is one that attempts to retrieve AOD from MODIS (Moderate Resolution Imaging Spectroradiometer) observations over bright surfaces. We took a different approach by using AOD retrievals from the MISR (Multiangle Imaging Spectroradiometer) instrument, which are robust to surface brightness, and by removing biased AOD retrievals from both MODIS and MISR. We found little bias (< 20%) between AERONET (AErosol RObotic NETwork) and our combined satellite AOD in sub-Saharan Africa. Although our PM_2.5_ estimates over sub-Saharan Africa (van Donkelaar et al. 2010) are subject to uncertainty, recent PM_2.5_ measurements in Ghana (Dionisio et al. 2010) indicate that Saharan dust is a significant regional source of PM_2.5_ and that our estimates may in fact be too low, both in contrast with Kumar’s expectations. We welcome additional *in situ* measurements for future comparisons.

The combination of satellite observations and CTMs offers great potential. The approach we presented in our article (van Donkelaar et al. 2010) took advantage of the fine resolution and observational nature of satellite AOD retrievals and estimates ground-level PM_2.5_ using the physically based framework of a CTM. Empirical methods, such as proposed by Kumar, can be effective over regions where sufficient surface measurements are available to train empirical (or semi-empirical) models. However, sufficient *in situ* measurements do not exist for most of the world, thus limiting the geographic scope of any method that is too dependent upon them. Expansion of the current global ground-based aerosol measurement network would provide a valuable data set to evaluate and improve the ability of CTMs to capture the AOD–PM_2.5_ relationship as well as the quality of the resultant satellite-based PM_2.5_ estimate.
